# Droplet epitaxy of InGaN quantum dots on Si (111) by plasma-assisted molecular beam epitaxy

**DOI:** 10.1186/s11671-023-03844-2

**Published:** 2023-04-07

**Authors:** Nurzal Nurzal, Ting-Yu Hsu, Iwan Susanto, Ing-Song Yu

**Affiliations:** 1 Department of Materials Science and Engineering, National Dong Hwa University, Hualien, 97401 Taiwan; 2 Department of Mechanical Engineering, Institut Teknologi Padang, Kp Olo Padang, 25143 Indonesia; 3 Department of Mechanical Engineering, Politeknik Negri Jakarta, Depok, 16424 Indonesia

**Keywords:** InGaN, Quantum dots, Molecular beam epitaxy, Droplet epitaxy, Photoluminescence

## Abstract

The droplet epitaxy of indium gallium nitride quantum dots (InGaN QDs), the formation of In–Ga alloy droplets in ultra-high vacuum and then surface nitridation by plasma treatment, is firstly investigated by using plasma-assisted molecular beam epitaxy. During the droplet epitaxy process, in-situ reflection high energy electron diffraction patterns performs the amorphous In–Ga alloy droplets transform to polycrystalline InGaN QDs, which are also confirmed by the characterizations of transmission electron microscopy and X-ray photoelectron spectroscopy. The substrate temperature, In–Ga droplet deposition time, and duration of nitridation are set as parameters to study the growth mechanism of InGaN QDs on Si. Self-assembled InGaN QDs with a density of 1.33 × 10^11^ cm^−2^ and an average size of 13.3 ± 3 nm can be obtained at the growth temperature of 350 °C. The photoluminescence emissions of uncapped InGaN QDs in wavelength of the visible red (715 nm) and infrared region (795 and 857 nm) are observed. The formation of high-indium composition of InGaN QDs via droplet epitaxy technique could be applied in long wavelength optoelectronic devices.

## Introduction

Indium gallium nitride (InGaN), one of the compound semiconductor materials, has been applied extensively in optoelectronics. For instance, InGaN quantum dots (QDs) have excellent characteristics for optoelectronic devices, such as light emitting diodes (LEDs) [1–3], infrared photodetectors [4–6], and laser diodes [7–9]. The most commonly used mechanism for the growth of self-assembly QDs is Stranski–Krastanov (SK) mode [10–12] through molecular beam epitaxy (MBE) [13, 14] and metal–organic chemical deposition (MOCVD) [15–17]. However, the SK mode has drawbacks as the growth of InGaN QDs triggered by layer strain, which can produce threading dislocations or defects, and uncontrollable density [10, 18]. On the other hand, the growth of crystal InGaN QDs with indium-rich composition and low-dislocation density is still challenging to broaden the emissions spectra from the visible red to the near-infrared regions [19]. Droplet epitaxy (DE) could be an alternative method to solve this problem for fabricating semiconductors' QDs [20, 21]. This is because DE technique has several advantages for the crystal growth of QDs, such as the strain-independent materials, lower temperature process, lattice-mismatched substrates, and the QDs growth without a wetting layer [22]. Some fundamental aspects in controlling the dimensions and density of GaN and InN QDs by DE have been investigated, such as the growth temperature, droplet deposition time, and the duration of nitridation [23, 24]. So far, there seems little or no report on the formation of InGaN QDs via the DE technique.

In this work, InGaN QDs have been fabricated on Si (111) substrate by forming In–Ga alloy droplets and then the nitridation process in the plasma-assisted molecular beam epitaxy (PA-MBE) system. The growth parameters of substrate temperature, deposition time of In–Ga droplets, and plasma nitridation duration have been adjusted to investigate the growth mechanism of InGaN QDs by DE. Some characterization tools were employed to analyze the morphology, chemical composition, crystallinity, and optical properties of InGaN QDs. This report demonstrates the growth of InGaN QDs by DE and the potential applications in long-wavelength optoelectronic devices.

## Methods

### Substrate preparation

A 2-inch silicon (111) wafer as a substrate was cleaned with acetone for 10 min and rinsed with deionized (DI) water in the ultrasonic oscillator. After that, the substrate was soaked in 10% hydrofluoric acid for 10 min and washed with DI water. Finally, the substrate was blown-dry with nitrogen gas and then immediately put into the load-lock chamber of our ULVAC PA-MBE system.

### InGaN QDs growth

Before the growth, the base pressure of growth chamber in PA-MBE system was ~ 4 × 10^7^ Pa. The Si substrate was thermally cleaned in the growth chamber at 600 °C for 10 min. The substrate was conducted the pre-nitridation process under 500 W RF plasma with 0.8 sccm high purity nitrogen for 30 min. Two source K-cells, filled with indium (6N) and gallium (6N), respectively, were maintained at the temperature of 800 °C. The beam equivalent pressures of In and Ga K-cells were 1.13 × 10^5^ and 8 × 10^6^ Pa, respectively. Different substrate temperatures (350, 400, and 450 °C) were set before the growth. For droplet epitaxy, the K-cells supplied In and Ga droplets for 30, 45, and 60 s. And then, the plasma nitridation process for those droplets on the surface for 0, 15, 30, and 45 min, respectively. The detailed parameters for the droplet epitaxy of InGaN QDs are listed in Table 1. The growth parameters include the substrate temperature of growth, the time of In–Ga droplet deposition, and the duration of plasma nitridation.

**Table 1:** Sample growth parameters for droplet epitaxy of InGaN QDs

Parameters	A1	A2	B1	B2	B3	C1	C2
Substrate		Si (111)					
Thermal cleaning		600 °C for 10 min					
Pre-nitridation		600 °C for 30 min					
Growth temperature (°C)	350	450	400	400	400	400	400
In/Ga deposition time (sec)	30	30	30	30	30	45	60
Nitridation duration (min)	30	30	0	15	45	30	30
K-cell temperature (°C)	800	800	800	800	800	800	800

### InGaN QDs characterizations

In the growth chamber of MBE, an in-situ 20 kV reflection high energy electron diffraction (RHEED) was used to monitor the surface condition of samples and the growth of InGaN QDs. After the growth, the surface morphology was observed by JEOL JSM-7000F field emission scanning electron microscopy (FE-SEM). Surface roughness and local contact voltage as a work function of the material were measured by Nanosurf C3000, including atomic force microscopy (AFM) and Kevin probe force microscopy (KPFM). The chemical bonding state and composition of InGaN QDs were determined by VGS Thermo K-alpha X-ray photoelectron spectroscopy (XPS). The microstructure of InGaN QDs was identified by JEOL JEM-3010 transmission electron microscopy (TEM) with energy-dispersive X-ray spectroscopy (EDX). The photoluminescence (PL) spectra of the InGaN QDs were obtained in the range of 530 and 900 nm, equipped with a 500 μW laser diode in the wavelength of 450 nm.

## Results and discussion

**Fig. 1:**
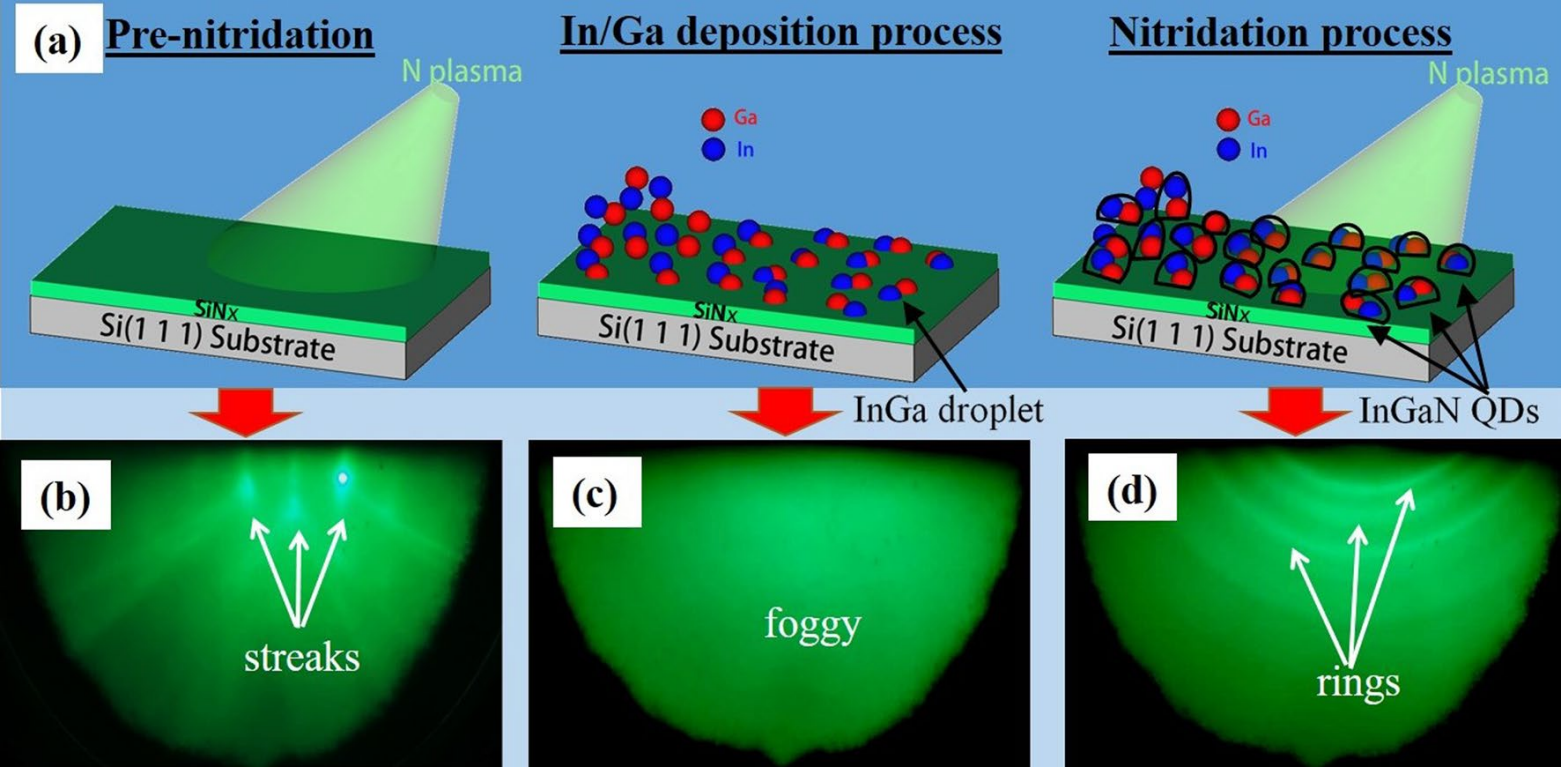
**a** Illustration of InGaN QDs growth on the Si (111) substrate via droplet epitaxy, **b** RHEED pattern of substrates after pre-nitridation at 600 °C for 30 min, **c** RHEED pattern after In–Ga deposition, and **d** RHEED pattern of InGaN QDs for B3 sample

During the growth of InGaN QDs, there are three main stages: pre-nitridation of Si substrate, In–Ga droplet deposition, and the formation of InGaN QDs. The schematic of the process is illustrated in Fig. 1a, which corresponds with the RHEED patterns in Fig. 1b–d, respectively. After the pre-nitridation process in Fig. 1b, the streaky RHEED pattern with Kikuchi lines indicates the flat surface formed with a crystalline structure [10, 14, 25]. A very thin SiN_x_ layer was formed because nitrogen atoms absorbed on the Si surface, which will also be reported by the observation of HR-TEM. After In–Ga droplet deposition at the growth temperature, the RHEED pattern in Fig. 1c became foggy. It reveals that the deposition of In–Ga alloy droplets is an amorphous structure [25]. In and Ga atoms from K-cells impinged, adsorbed, and migrated on the surface. After following exposure to nitrogen plasma, the In–Ga droplets grew and completely crystallized into InGaN QDs. Meanwhile, the RHEED pattern changed from foggy to ring-type, as shown in Fig. 1d, which performed the formation of polycrystalline InGaN QDs on the SiNx/Si surface [24].

**Fig. 2:**
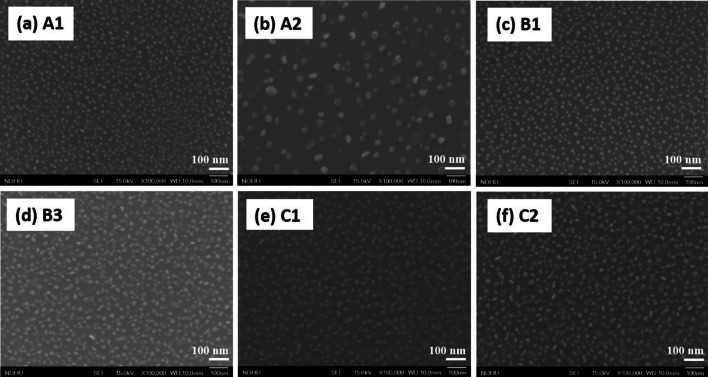
FE-SEM images of InGaN QDs on Si (111) substrate: **a** A1, **b** A2, **d** B3, **e** C1 and **f** C2; In–Ga droplets on Si(111) substrate: **c** B1

FE-SEM was used to observe InGaN QDs and the surface morphologies of all samples, shown in Fig. 2. The average lateral dimension and density of InGaN QDs on Si can be calculated from the images, which are summarized in Table 2. Three different growth parameters will be discussed. First, Fig. 2a, b shows FE-SEM images of A1 and A2 samples, respectively. The higher growth temperature increased the average diameter of InGaN QDs from 13.3 ± 3.0 to 28.7 ± 13.2 nm, and the dot density decreased dramatically from 1.33 × 10^11^ to 1.66 × 10^10^ cm^−2^. These can be attributed to the improved migration length and aggregation of adatoms to form bigger dots at higher growth substrate temperatures [14]. The evaporation of indium from the substrate at higher growth temperature also occurred during the growth, which could significantly decrease the dot density. In addition, the higher standard deviation of dot diameter (28.7 ± 13.2 nm) was observed for sample A2, grown at 450 °C. It is because of the Ostwald ripening phenomenon, which made the In–Ga droplets bigger and inhomogeneous InGaN QDs after nitridation. Both nanostructures of InGaN quantum dots and rings can be observed in this sample, shown in Fig. 2b. Second, when the duration of plasma nitridation increased from 0 to 45 min, images are shown in Fig. 2c for sample B1 and in Fig. 2d for sample B3, respectively. The average diameter of InGaN QDs increased from 14.7 ± 2.2 to 24.8 ± 8.2 nm. For sample B1 (without nitridation), In–Ga alloy droplets on the surface performed in a round shape. After the nitridation process for 45 min, sample B3, InGaN QDs in irregular shapes were observed, and the standard deviation of dot diameter increased. During the nitridation process, the nearby small dots could diffuse together to form bigger dots. The longer nitriding time supplied more active nitrogen radicals to bond with In and Ga adatoms to form InGaN QDs [20]. The dot density reduced from 1.11 × 10^11^ to 1.0 × 10^11^ dots/cm^2^. Third, the different deposition time of In–Ga droplet was investigated. In Fig. 2e, f, the deposition time from 45 to 60 s made the dot diameter get bigger from 22.4 ± 6.3 to 23.5 ± 8.6 nm, and also increased dot densities from 8.51 × 10^10^ to 8.96 × 10^10^ dots/cm^2^. The longer In–Ga deposition time supplied more atoms to get a larger dot size and higher density after the growth. The influence of In–Ga deposition time seems not prominent at fixed growth temperature and nitridation time.

**Table 2:** Average diameter and density of InGaN QDs or In–Ga droplets

Parameters	Growth temp		Nitridation duration			In–Ga time	
	A1	A2	B1	B2	B3	C1	C2
Diameter (nm)	13.3 ± 3.0	28.7 ± 13.2	14.7 ± 2.2	16.2 ± 5.7	24.8 ± 8.2	22.4 ± 6.3	23.5 ± 8.6
Density (dots/cm^2^)	1.33 × 10^11^	1.66 × 10^10^	1.11 × 10^10^	1.37 × 10^11^	10.0 × 10^10^	8.51 × 10^10^	8.96 × 10^10^

**Fig. 3:**
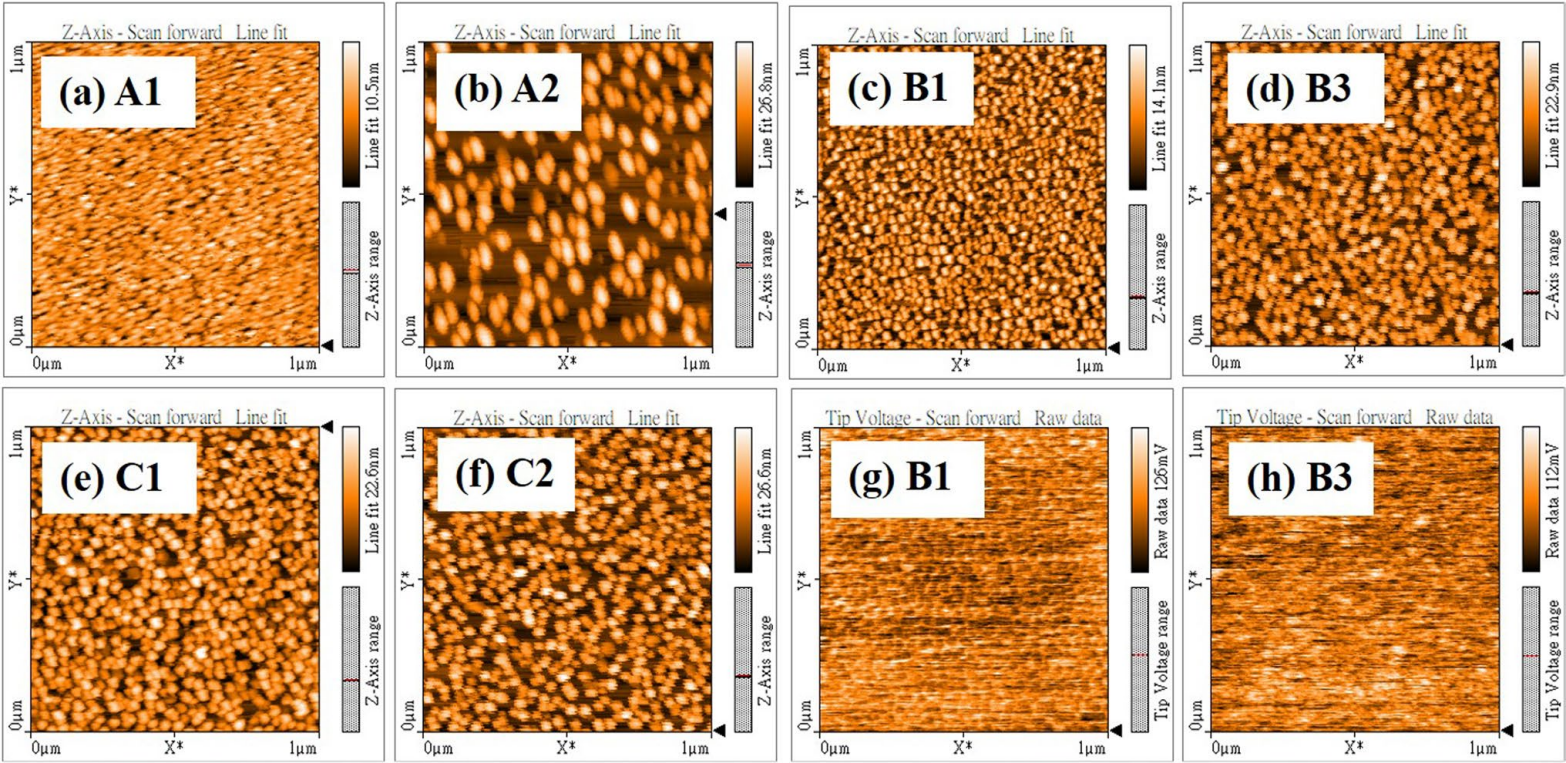
AFM images: **a** A1, **b** A2, **c** B1, **d** B3, **e** C1, and **f** C2; KPFM images: **g** B1, and **h** B3

Figure 3 shows AFM and KPFM images of InGaN QDs on the Si substrate with different growth parameters. The scan area of AFM measurement was set as 1 µm × 1 µm. Besides, KPFM was used to characterize the local contact potential difference (LCPD) as a work function between the AFM tip and the sample surfaces of InGaN QDs [26, 27]. The InGaN QDs can be observed in the AFM images in Fig. 3, and the average surface roughness can be calculated in Table 3. The size of InGaN QDs and surface roughness increased with higher growth temperature, which is consistent with the results of SEM observation. InGaN QDs grown at 350 °C had a smaller average diameter and performed a smaller surface roughness of 1.27 nm, as presented in Fig. 3a. When the growth temperature was increased to 450 °C, the diameter of InGaN QDs became three times bigger, and the average surface roughness increased to 4.67 nm, shown in Fig. 3b. Meanwhile, the surface potential decreased from 397.02 to 243.40 mV. As the nitridation duration increased from 0 to 45 min, shown in Fig. 3c, d, bigger dots were obtained, and surface roughness increased from 2.57 to 3.82 nm. Interestingly, LCPD values of B1 and B3 decreased dramatically from 680.11 to 365.9 mV in Fig. 3g, h. It can evidence that the nitridation process of the DE technique made In–Ga alloy droplets transform to InGaN QDs. Moreover, as the growth at higher temperature or a prolonged nitridation time, the average LCPD values decreased, which means the surface potential decreases with decreasing indium content of InGaN [28]. Finally, the deposition time of In–Ga droplets increased from 45 to 60 s, shown in Fig. 3e, f. The average roughness increased from 3.78 to 4.80 nm, which is consistent with the observation of SEM images.

**Table 3:** Root mean square (RMS) of AFM and local contact potential difference (LCPD) of KPFM for all samples

Parameters	Growth temp		Nitridation duration			In–Ga time	
	A1	A2	B1	B2	B3	C1	C2
RMS (nm)	1.27	4.67	2.57	3.33	3.83	3.79	4.80
LCPD (mV)	397.02	243.40	680.11	451.34	365.90	494.37	446.63

**Fig. 4:**
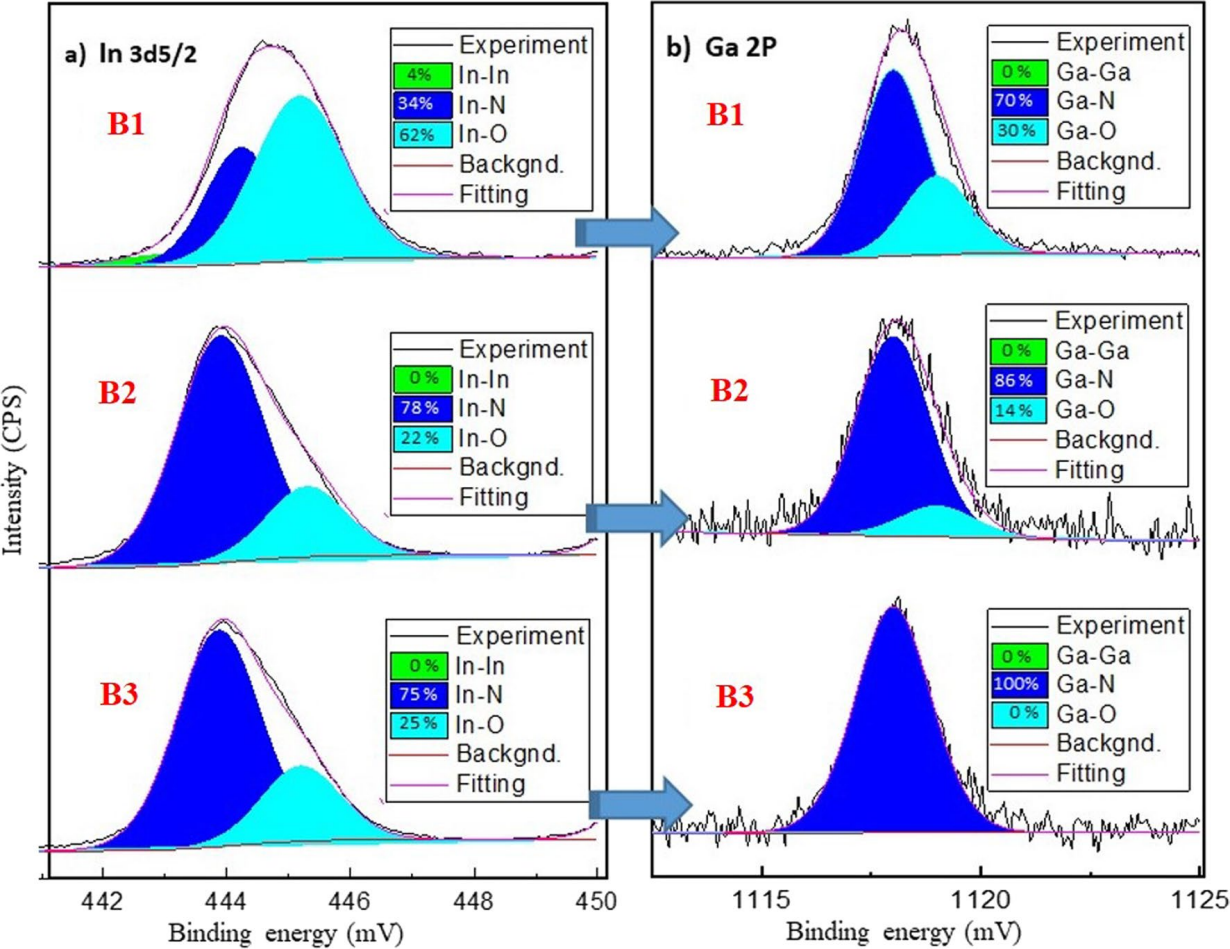
De-convoluted **a** In 3*d*_5/2_ and **b** Ga 2*p* XPS spectra for samples B1, B2, and B3

The surface chemical condition of InGaN QDs was measured by XPS, and the chemical composition of InGaN can be further analyzed by the fitting software. The de-convoluted XPS spectra for In 3*d*_5/2_ are presented in Fig. 4a with the fitting area of In–In, In–N, and In–O bonding for samples B1, B2, and B3. Similarly, Fig. 4b shows the spectra of Ga 2*p* with the fitting area of Ga–Ga, Ga–N, and Ga–O bonding. For sample B1 (without nitridation), the peak positions of binding energy for In–In, In–N, In–O, Ga–N, and Ga–O are around 443, 444.2, 445.2, 1118, and 1119 eV. The fitting areas of In 3*d*_5/2_ and Ga 2*p* with the binding for In–In, In–N, In–O, Ga–Ga, Ga–N, and Ga–O are 4, 34, 62, and 0, 70, 30%, respectively. The high content of In-O and Ga-O came from the absorption of oxygen and oxidation on the surface of In–Ga droplets for B1. When the nitridation duration increased for samples B2 and B3, more impinging nitrogen radicals with In–Ga adatoms and nitridation reaction made Ga–N and In–N bonds dominate [29]. Sample B3 can obtain 100% Ga–N bond and without any Ga–O bond. In short, XPS data in Fig. 4 also can evidence the nitridation reaction of InGaN QDs by droplet epitaxial growth. However, the In–O bonds still existed in B2 and B3 as 22 and 25%, respectively. It is because of the difference in formation enthalpy between GaN and InN, that could result in the segregation of indium clusters after droplet epitaxy [30].

The XPS data for all samples is summarized in Table 4. The fitting area can be used to roughly estimate the average chemical composition of InGaN on the surface without considering the phase separation effect [31]. To compare the parameter of growth temperature for samples A1 and A2, higher growth temperatures made indium composition redue from 82 to 60%, but increased gallium from 18 to 40%. At higher growth temperature, Ga atoms are more accessible to absorb, diffuse and react with nitrogen than In atoms. However, the phase separation, InN decomposition, and In desorption can occur at higher temperatures [32]. In short, the substrate temperature is also an important parameter for InGaN QDs with high-indium content by droplet epitaxy.

**Table 4:** The peak position, fitting area, and average surface chemical composition for all samples

Measurement	In 3*d* 5/2			Ga 2*p*			N 1s		
	In–In	In–N	In–O	Ga-Ga	Ga–N	Ga–O	N–In	N–Ga	N–O
Peak (eV)	0	443.96	445.4	0	1118	119	396.5	97.5	398.9
Area	0	22,157	8756	0	3621.2	486.2	2723.7	1688	223.5
A1, surface compositions: *x*(In) = 0.82, 1−*x*(Ga) = 0.18								In_0.82_Ga_0.18_N	
Peak (eV)	0	443.96	445.3	0	1118	1119	396.5	97.4	398.9
Area	0	10,981	6049.7	0	6171.1	513.6	2390.2	853.9	991.8
A2, surface compositions: *x*(In) = 0.60, 1−*x*(Ga) = 0.40								In_0.60_Ga_0.40_N	
Peak (eV)	443	444.2	445.2	0	118	1119	396.3	97.3	398.9
Area	1 161.5	8651.4	15,588	0	9203.3	3945.5	981.43	732.2	1079.2
B1, surface compositions: *x*(In) = 0.48, 1−*x*(Ga) = 0.52								In_0.48_Ga_0.52_	
Peak (eV)	0	443.9	445.3	0	1118	1119	396.2	397.5	398.9
Area	0	29,469	884,230	0	2814.9	448.9	2719.2	1262.4	265
B2, surface compositions: *x*(In) = 0.89, 1−*x*(Ga) = 0.11								In_0.89_Ga_0.11_N	
Peak (eV)	0	443.87	445.2	0	1118	0	396.2	397.4	398.7
Area	0	33,373	10,885	0	5073.6	0	2769.4	2241.2	352.6
B3, surface compositions: *x*(In) = 0.83, 1−*x*(Ga) = 0.17								In_0.83_Ga_0.17_N	
Peak (eV)	0	443.70	444.90	0	1118	1118.9	396.4	397.4	398.9
Area	0	26,909	14,581	0	5024.2	1015.2	4534.6	2322.9	357.7
C1, surface compositions: *x*(In) = 0.81, 1−*x*(Ga) = 0.19								In_0.81_Ga_0.19_N	
Peak (eV)	0	444.2	445.2	0	1118.1	1119	396.5	397.5	398.6
Area	0	20,163	21,337	0	3182.2	3313.8	2572.9	2166.4	1050.1
C2, surface compositions: *x*(In) = 0.81, 1−*x*(Ga) = 0.19								In_0.81_Ga_0.19_N	

**Fig. 5:**
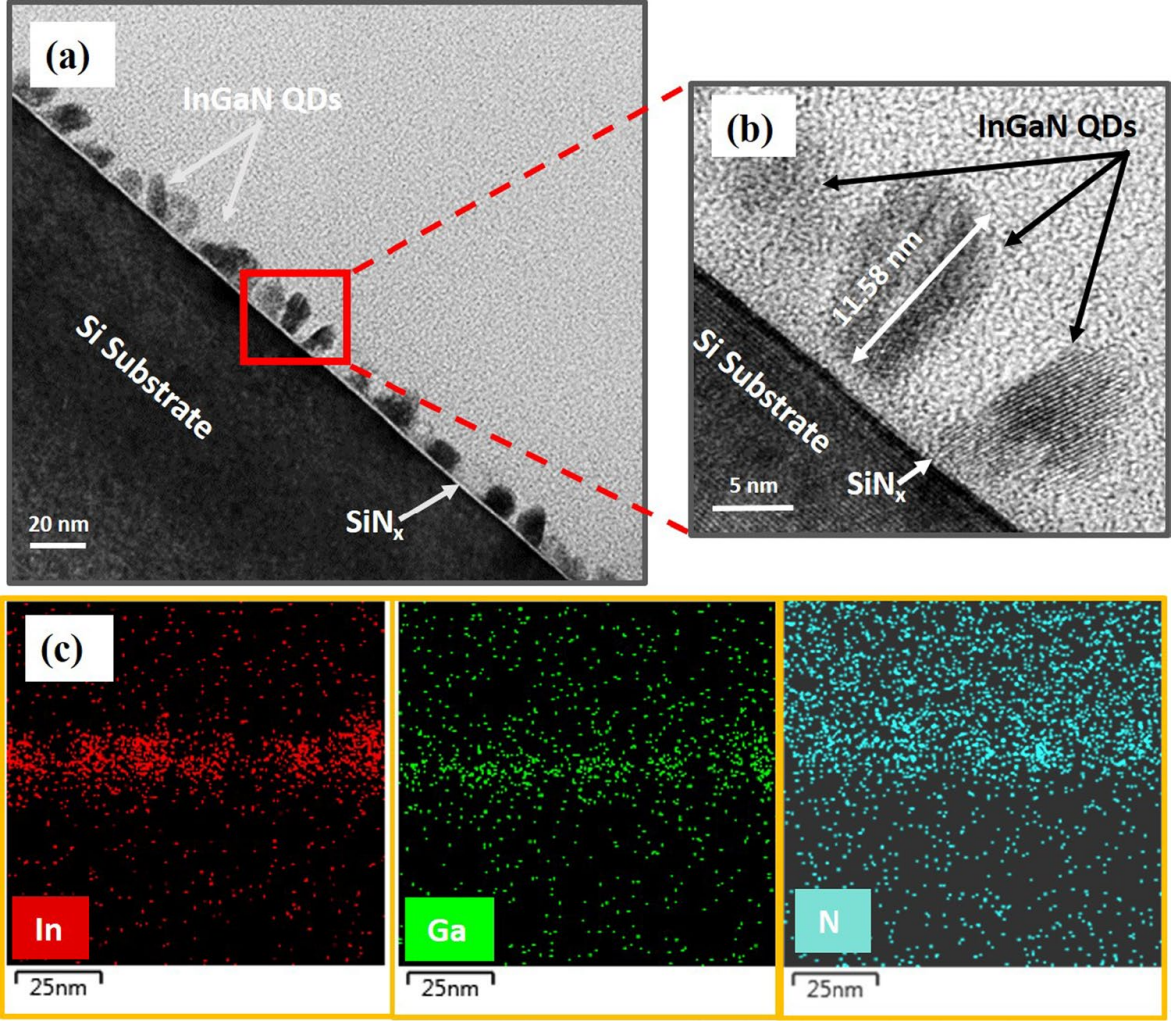
**a** Cross-sectional TEM image of InGaN QDs grown on the Si (111) substrate for sample B3, **b** High-resolution TEM image for the red rectangular area, and **c** EDX mapping on the elements of indium, gallium, and nitrogen

InGaN QDs have been observed by TEM after the preparation of sample B3 by focused ion beam technique. In Fig. 5a of the cross-sectional TEM image, self-assembled InGaN QDs have been grown on the surface of Si substrate by the growth mode of droplet epitaxy. Figure 5b shows the enlargement of the red rectangular area in Fig. 5a by the high-resolution TEM image. Three InGaN nanodots were observed, and the one in the center has a height of 11.58 nm. The dot at the lower right corner of Fig. 5b obviously performs a single crystal structure of InGaN. A very thin amorphous SiNx layer between the Si substrate and InGaN QDs can be observed, due to the pre-nitridation treatment on the Si substrate [33]. Moreover, the chemical composition analysis was conducted by EDX spectral mapping on elements In, Ga, and N, presented in Fig. 5c, respectively, which also could confirm the droplet epitaxial growth of InGaN QDs.

**Fig. 6:**
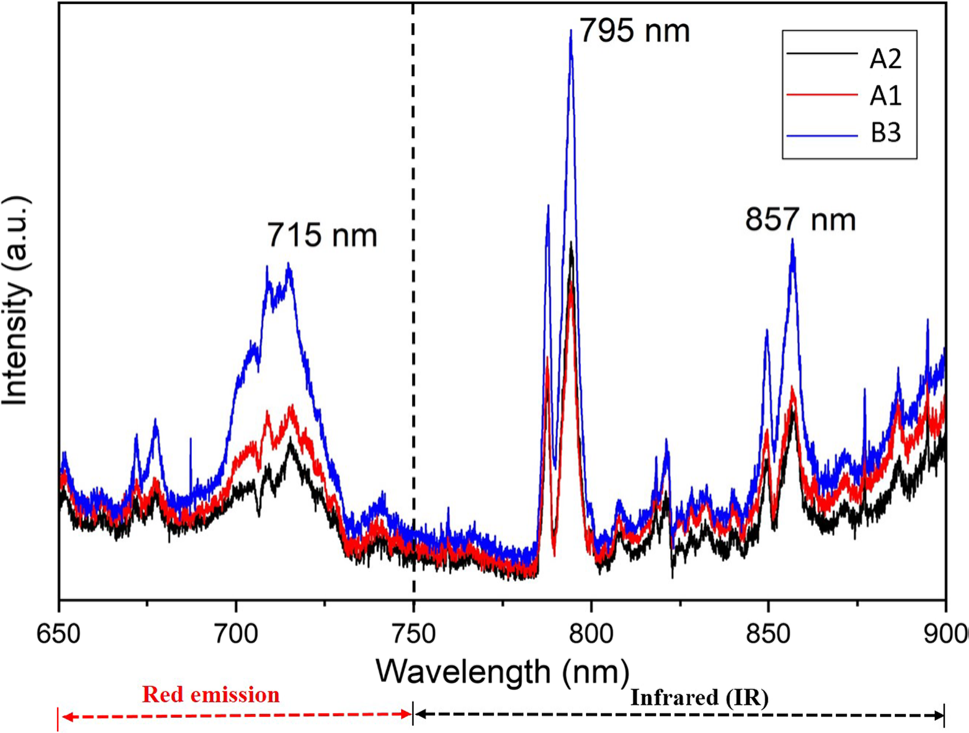
PL spectra at the temperature of 80 K for A1, A2, and B3 samples

The optical properties of uncapped InGaN QDs for samples A1, A2, and B3 were characterized by PL spectroscopy measured at the temperature of 80 K. Figure 6 shows three major emission peaks around the wavelength of 715, 795 and 857 nm, and the emissions of InGaN QDs are in the wavelength of visible red and infrared region. The broad emission peaks indicated the inhomogeneous size, composition gradient, or random distribution of InGaN QDs [34]. The spiky PL peaks could be the noise from InGaN QDs without capping layer, which attributed to the surface recombination of carriers.

The bandgap of In_x_Ga_1−x_N alloy is in the range of 0.7–3.4 eV, depending on the composition of indium [35]. The emission spectra of InGaN QDs in the wavelength of visible red and infrared region could imply the high-indium composition of InGaN QDs. Among all the samples, B3, the sample with longer nitridation duration, exhibited the highest peak intensity of PL spectra. It indicates that the duration of nitridation could be the most important parameter for the growth of InGaN QDs via droplet epitaxy.

## Conclusions

The first investigation for the droplet epitaxial growth of InGaN QDs on Si (111) substrate was proposed by using the PA-MBE system. The growth of InGaN QDs was signed with the ring-type RHEED pattern, indicating the transformation from the amorphous In–Ga droplets to the polycrystalline InGaN structure. Moreover, high-resolution TEM data also proved the crystal growth of uncapped InGaN QDs. Three parameters, substrate temperature, duration of nitridation, and In–Ga deposition time were conducted to study the growth of InGaN QDs. During the droplet epitaxial growth, different substrate temperatures influenced adatoms’ adsorption, desorption, and surface diffusion to control the average size and density of InGaN QDs. The higher density of 1.33 × 10^11^ cm^−2^ and smaller size of 13.3 ± 3.0 nm can be obtained at the lower growth temperature of 350 °C. Then, the duration of plasma nitridation had less impact on the density of InGaN QDs, but it was an essential parameter in the formation of crystal InGaN QDs. The round-shape In–Ga alloy droplets became polygon of InGaN QDs. As the duration of nitridation increased, the surface chemical compositions of In–N and Ga–N bonds increased, and intensity of photoluminescence enhanced. It could be due to the crystal-quality improvement of InGaN QDs. The emissions of InGaN QDs were in wavelength of the visible red and infrared region, indicating the formation of the high-indium composition of InGaN QDs via droplet epitaxy technique.

## Data Availability

The datasets generated during and/or analyzed during the current study are available from the corresponding author on reasonable request.

## References

[1] Chung J-Y, Li Z, Goodman SA, So J, Syaranamual GJ, Mishra TP (2021). Light-emitting V-pits: an alternative approach toward luminescent indium-rich InGaN quantum dots. ACS Photonics.

[2] Park D, Min D, Nam O (2017). Growth mechanism of InGaN nanodots on three-dimensional GaN structures. Phys Status Solidi: Rapid Res Lett.

[3] Figge S, Tessarek C, Aschenbrenner T, Hommel D (2011). Ingan quantum dot growth in the limits of stranski-krastanov and spinodal decomposition. Phys Status Solidi Basic Res.

[4] Aiello A, Hasibul Hoque AKM, Baten MZ, Bhattacharya P (2019). High-gain silicon-based InGaN/GaN dot-in-nanowire array photodetector. ACS Photonics.

[5] Seo H, Park JH, Kwon OH, Kwon OP, Kwak SK, Kim SW (2020). Highly qualified InP based QDs through a temperature controlled ZnSe shell coating process and their DFT calculations. Nanoscale Adv.

[6] Vichi S, Bietti S, Khalili A, Costanzo M, Cappelluti F, Esposito L (2020). Droplet epitaxy quantum dot based infrared photodetectors.

[7] Yin H, Qian Y, Xie L, Song C, Wang X, Chen H (2019). Electrocatalytic activity of InN/InGaN quantum dots. Electrochem commun.

[8] Chen P, Chua SJ, Tan JN (2006). High-density InGaN nanodots grown on pretreated GaN surfaces. Appl Phys Lett.

[9] Woodward JM, Nikiforov AY, Ludwig KF, Moustakas TD (2017). Analysis of InGaN nanodots grown by droplet heteroepitaxy using grazing incidence small-angle X-ray scattering and electron microscopy. J Appl Phys.

[10] Wang L, Wang L, Yu J, Hao Z, Luo Y, Sun C (2019). Abnormal Stranski–Krastanov mode growth of green InGaN quantum dots: morphology, optical properties, and applications in light-emitting devices. ACS Appl Mater Interfaces.

[11] Qian Y, Wang P, Rao L, Song C, Yin H, Wang X (2020). Electric dipole of InN/InGaN quantum dots and holes and giant surface photovoltage directly measured by Kelvin probe force microscopy. Sci Rep.

[12] Linares-García G, Meza-Montes L, Stinaff E, Alsolamy SM, Ware ME, Mazur YI (2016). Optical properties of a quantum dot-ring system grown using droplet epitaxy. Nanoscale Res Lett.

[13] Chin CW, Hassan Z, Yam FK, Ahmad MA (2013). Growth of self-assembled InGaN quantum dots on Si (111) at reduced temperature by molecular beam epitaxy. Thin Solid Films.

[14] Zhang X, Wenxian Y, Xing Z, Qiu H, Ying G, Bian L (2021). investigation of micromorphology and carrier recombination molecular beam epitaxy. Cryst Artic.

[15] Dong H, Qu K, Liang J, Zhang A, Jia Z, Jia W (2020). Evolution mechanism of InGaN quantum dots and their optical properties. Opt Mater (Amst).

[16] Liu G, Zhao H, Zhang J, Park JH, Mawst LJ, Tansu N (2011). Selective area epitaxy of ultra-high density InGaN quantum dots by diblock copolymer lithography. Nanoscale Res Lett.

[17] Yu L, Wang L, Yang P, Hao Z, Yu J, Luo Y (2022). Metal organic vapor phase epitaxy of high-indium-composition InGaN quantum dots towards red micro-LEDs. Opt Mater Express.

[18] Prabakaran K, Ramesh R, Arivazhagan P, Jayasakthi M, Sanjay S, Surender S (2022). Effect of spiral-like islands on structural quality, optical and electrical performance of InGaN/GaN heterostructures grown by metal organic chemical vapour deposition. Mater Sci Semicond Process.

[19] Um DY, Ra YH, Park JH, Hong GE, Lee CR (2021). Near-IR emission of InGaN quasi-quantum dots on non-polar GaN nanowire structures. Nanoscale Adv.

[20] Aseev P, Gačević Z, Mánuel JM, Jiménez JJ, García R, Morales FM (2018). Formation mechanisms of single-crystalline InN quantum dots fabricated via droplet epitaxy. J Cryst Growth.

[21] Azadmand M, Barabani L, Bietti S, Chrastina D, Bonera E, Acciarri M (2018). Droplet controlled growth dynamics in molecular beam epitaxy of nitride semiconductors. Sci Rep.

[22] Sanguinetti S, Bietti S, Koguchi N. Droplet epitaxy of nanostructures. In: Molecular beam epitaxy. Elsevier Inc; 2018. pp 293–314.

[23] Yu IS, Chang CP, Yang CP, Lin CT, Ma YR, Chen CC (2014). Characterization and density control of GaN nanodots on Si (111) by droplet epitaxy using plasma-assisted molecular beam epitaxy. Nanoscale Res Lett.

[24] Chen HJY, Yang DL, Huang TW, Yu IS (2016). Formation and temperature effect of InN nanodots by PA-MBE via droplet epitaxy technique. Nanoscale Res Lett.

[25] Hasegawa S. Reflection high-energy electron diffraction. In: Characterization of Materials. Hoboken: John Wiley & Sons, Inc.; 2012. p. 1928–31. 10.1002/0471266965.com139.

[26] Melitz W, Shen J, Kummel AC, Lee S (2011). Kelvin probe force microscopy and its application. Surf Sci Rep.

[27] Salerno M, Dante S (2018). Scanning Kelvin probe microscopy: Challenges and perspectives towards increased application on biomaterials and biological samples. Materials (Basel).

[28] Kimura T, Fukumoto E, Yamaguchi T, Wang K, Kaneko M, Araki T (2011). Investigation of InN mole fraction fluctuation in InGaN films grown by RF-MBE. Phys Status Solidi Curr Top Solid State Phys.

[29] Tian M, Qian YD, Zhang C, Li L, Yao SD, Ferguson IT (2018). Investigation of high indium-composition InGaN/GaN heterostructures on ZnO grown by metallic organic chemical vapor deposition. Opt Mater Express.

[30] Doppalapudi D, Basu SN, Ludwig KF, Moustakas TD (1998). Phase separation and ordering in InGaN alloys grown by molecular beam epitaxy. J Appl Phys.

[31] Kumar P, Rodriguez PEDS, Gómez VJ, Alvi NH, Calleja E, Nötzel R (2013). First demonstration of direct growth of planar high-in-composition InGaN layers on Si. Appl Phys Express.

[32] Tessarek C, Figge S, Aschenbrenner T, Bley S, Rosenauer A, Seyfried M, Kalden J, Sebald K, Gutowski J, Hommel D (2011). Strong phase separation of strained InxGa_1−x_N layers due to spinodal and binodal decomposition. Phys Rev B.

[33] Aseev P, Rodriguez PEDS, Kumar P, Gómez VJ, Alvi NUH, Mánuel JM (2013). Uniform low-to-high in composition InGaN layers grown on Si. Appl Phys Express.

[34] Chen P, Chen A, Chua SJ, Tan JN (2007). Growth and optical properties of highly uniform and periodic InGaN nanostructures. Adv Mater.

[35] Kour R, Arya S, Verma S, Singh A, Mahajan P, Khosla A (2020). Review-recent advances and challenges in indium gallium nitride (In_x_Ga_1-x_N) Materials for solid state lighting. ECS J Solid State Sci Technol.

